# Supplementary material for: Polyketide synthase gene clusters in the ericoid mycorrhizal fungus Oidiodendron maius: genome-wide prediction, expression profiling, and functional characterization of knockout mutants
Source: Front Plant Sci. 2026 Sep 16;17:1924430. doi: 10.3389/fpls.2026.1924430 (PMC13623903; doi:10.3389/fpls.2026.1924430)
Supplement: Supplementary file 1 [file SupplementaryFile1.zip › Supplementary Materials/Supplementary Folder 1.zip!/Oidma1_AssemblyScaffolds_Repeatmasked/knownclusterblast/cluster53/jgi.p_Oidma1_173323_mibig_hits.html]

| MIBiG Protein | Description | MIBiG Cluster | MiBiG Product | % ID | % Coverage | BLAST Score | E-value |
| --- | --- | --- | --- | --- | --- | --- | --- |
| ALI92656.1 | MRR1\_Major\_Facilitator\_Superfamily\_(MFS)\_protein | BGC0001338 | Citrinin\_biosynthetic\_gene\_cluster | 59 | 98.1963927856 | 566 | 4.4e-161 |
| ABK64181.1 | MFS\_transporter | BGC0001247\_c4 | Cercosporin\_biosynthetic\_gene\_cluster | 44 | 94.1883767535 | 407 | 2e-113 |
| EED18004.1 | conserved\_hypothetical\_protein | BGC0000154 | Stipitatic\_acid\_biosynthetic\_gene\_cluster | 43 | 92.1843687375 | 393 | 3e-109 |
| EWG54280.1 | hypothetical\_protein | BGC0001190 | Fusaric\_acid\_biosynthetic\_gene\_cluster | 43 | 93.1863727455 | 356 | 5.2e-98 |
| CCH26293.1 | putative\_MFS-type\_transporter | BGC0001158\_c4 | Bikaverin\_biosynthetic\_gene\_cluster | 39 | 92.7855711423 | 346 | 4.1e-95 |
| CCT67996.1 | bikaverin\_cluster-efflux\_pump | BGC0000030 | Bikaverin\_biosynthetic\_gene\_cluster | 39 | 94.1883767535 | 345 | 9.2e-95 |
| KFH48706.1 | putative\_MFS-type\_transporter-like\_protein | BGC0000317 | Cephalosporin\_biosynthetic\_gene\_cluster | 41 | 91.3827655311 | 338 | 1.5e-92 |
| CAP95407.1 |  | BGC0001404 | Sorbicillin\_biosynthetic\_gene\_cluster | 38 | 96.7935871743 | 336 | 4.3e-92 |
| ACD39772.1 | major\_facilitator\_superfamily\_transporter | BGC0000134 | Radicicol\_biosynthetic\_gene\_cluster | 37 | 96.1923847695 | 315 | 1e-85 |
